# Attitudes and Behaviors towards Food and Weight in Late Pregnancy: A Comparative Approach between Individuals with and without Previous Bariatric Surgery

**DOI:** 10.3390/healthcare12030342

**Published:** 2024-01-30

**Authors:** Chloé Caredda, Audrey St-Laurent, Marianne Gagnon, Stéphanie Harrison, Emilie Bernier, Geneviève Gagnon, Anne-Sophie Plante, Simone Lemieux, Catherine Bégin, Simon Marceau, Laurent D. Biertho, André Tchernof, Véronique Provencher, Vicky Drapeau, Andréanne Michaud, Anne-Sophie Morisset

**Affiliations:** 1 Centre Nutrition, Santé et Société (NUTRISS), Institut sur la Nutrition et les Aliments Fonctionnels (INAF), Université Laval, Québec, QC G1V 0A6, Canadaaudrey.st-laurent@crchudequebec.ulaval.ca (A.S.-L.); marianne.gagnon.12@ulaval.ca (M.G.); stephanie.harrison.1@ulaval.ca (S.H.); emilie.bernier.4@ulaval.ca (E.B.); anne-sophie.plante@crchudequebec.ulaval.ca (A.-S.P.); simone.lemieux@fsaa.ulaval.ca (S.L.); catherine.begin@psy.ulaval.ca (C.B.); veronique.provencher@fsaa.ulaval.ca (V.P.); vicky.drapeau@kin.ulaval.ca (V.D.); andreanne.michaud@fsaa.ulaval.ca (A.M.); 2 Axe Endocrinologie et Néphrologie, Centre de Recherche du CHU de Québec, Université Laval, Québec, QC G1V 4G2, Canada; 3 École de Nutrition, Université Laval, Québec, QC G1V 0A6, Canada; genevieve.gagnon.13@ulaval.ca (G.G.); andre.tchernof@criucpq.ulaval.ca (A.T.); 4 École de Psychologie, Université Laval, Québec, QC G1V 0A6, Canada; 5 Axe Obésité, Diabète de Type 2 et Métabolisme, Centre de Recherche de l’Institut Universitaire de Cardiologie et de Pneumologie de Québec, Québec, QC G1V 4G5, Canada; simon.marceau@fmed.ulaval.ca (S.M.); laurent.biertho@criucpq.ulaval.ca (L.D.B.); 6 Département de Kinésiologie, Université Laval, Québec, QC G1V 0A6, Canada

**Keywords:** bariatric surgery, pregnancy, eating behaviors, attitudes towards weight gain, intuitive eating

## Abstract

The aims of this study were to compare, between pregnant individuals with and without bariatric surgery: (1) eating behaviors, (2) intuitive eating components and, (3) attitudes towards weight gain. This retrospective study included data collected in healthy pregnant individuals with and without previous bariatric surgery who were recruited at the *Centre Hospitalier Universitaire (CHU) de Québec-Université Laval*. Pregnant individuals who underwent bariatric surgery (biliopancreatic bypass with duodenal switch [n = 14] or sleeve gastrectomy [n = 5]) were individually matched, for age (±0.4 years) and body mass index (BMI) (±0.3 kg/m^2^), with pregnant individuals who have not received bariatric surgery. In the second trimester, participants completed the Three Factor Eating Questionnaire (TFEQ) and the Intuitive Eating Scale 2 (IES-2). In the third trimester, participants completed the French version of the Pregnancy Weight Gain Attitude Scale assessing attitudes towards weight gain. Pregnant individuals who have had bariatric surgery had a higher score for flexible restraint and a lower score for situational susceptibility to disinhibition compared to individuals who have not had undergone bariatric surgery (2.89 ± 1.15 vs. 1.95 ± 1.31; *p* = 0.04 and 1.11 ± 1.29 vs. 2.79 ± 1.44, respectively; *p* < 0.001). Regarding intuitive eating, pregnant individuals who experienced bariatric surgery had a higher score for reliance on internal hunger and satiety cues and a lower one for unconditional permission to eat compared with those who had not experienced bariatric surgery (3.99 ± 0.81 vs. 3.30 ± 1.03; *p* = 0.02 and 3.28 ± 0.54 vs. 3.61 ± 0.68, respectively; *p* = 0.03). No difference in attitudes towards weight gain was observed between groups. Overall, pregnant individuals who had undergone bariatric surgery had different eating behaviors and intuitive eating components compared to pregnant individuals without bariatric surgery. These results need to be confirmed in further studies with larger sample sizes.

## 1. Introduction

Obesity can be defined as a chronic and progressive disease characterized by an excessive accumulation of adipose tissue that has an impact on metabolic health and quality of life of those affected [1,2]. Current treatment options, other than surgical intervention, are rarely effective in maintaining long-term weight loss and improving, or even resolving, the comorbidities associated with obesity in its severe form [1]. Indeed, bariatric surgery is the most effective treatment modality for severe obesity and its comorbidities, since it treats obesity-related comorbidities, such as type 2 diabetes, and also improves health and quality of life [3].

Bariatric surgery includes different procedures that can be classified as restrictive or restrictive and malabsorptive [4]. For example, sleeve gastrectomy (SG) is a restrictive procedure, while biliopancreatic bypass with duodenal switch (BPD-DS) and Roux-en-Y bypass (RYGB) are procedures that are both restrictive and malabsorptive. Individuals who have received sleeve gastrectomy, Roux-en-Y gastric bypass or biliopancreatic bypass with duodenal switch experienced important physical and metabolic changes that can be partially explained by changes in the gastrointestinal tract, thus resulting in a significant energy deficit balance and concurrent weight loss [3,5]. While bariatric surgery leads to immediate weight loss, long-term failure and weight regain is possible [6,7]. Indeed, a significant proportion of candidates seeking bariatric surgery exhibit suboptimal eating behaviors (e.g., disinhibition) prior to the surgery that may persist after the surgery and hinder long-term success [8,9]. However, little is known about eating behaviors and intuitive eating components in this context, as current recommendations focus primarily on diet quality and supplementation [10].

As for bariatric surgery, pregnancy itself also implies physical and metabolic changes. One of these changes is gestational weight gain (GWG), which can have an impact on body image, eating behaviors and attitudes towards food [11,12]. However, little is known about how eating behaviors, intuitive eating components and attitudes towards weight gain may vary during pregnancy. Indeed, studies that focused on eating behaviors and attitudes during pregnancy have shown that, in late pregnancy, pregnant individuals with a positive attitude towards weight gain have healthier eating behaviors—i.e., they appear to be less restrictive and more intuitive [11]—and that pregnant individuals who eat more intuitively have adequate GWG in the first trimester [12].

Among the patients undergoing bariatric surgery, approximately three out of four identify as women, of whom more than half are of childbearing age and therefore could be pregnant in the years following surgery [13,14,15]. In those individuals, a pregnancy after a bariatric surgery, regardless of type, presents lower risk for gestational diabetes mellitus or large-for-gestational-age neonates, compared to pregnant obese individuals who never had bariatric surgery [13,16,17]. Nevertheless, to the best of our knowledge, the effect of bariatric surgery on eating behaviors, intuitive eating components and attitudes towards weight gain, in this specific population has not been studied yet. Therefore, individuals who have undergone bariatric surgery before pregnancy may have a unique relationship with food and weight, which means they will likely exhibit different eating behaviors and attitudes toward weight gain in comparison with those who have not had bariatric surgery. In that context, it would be highly relevant to study the potential differences in eating behaviors/attitudes between pregnant individuals post bariatric surgery and pregnant individuals without previous bariatric surgery.

Therefore, the main objective of the present study is to compare, between pregnant individuals with and without bariatric surgery: 1. eating behaviors, 2. intuitive eating components and, 3. attitudes towards weight gain. Our hypotheses were that, compared to pregnant individuals without a history of bariatric surgery, pregnant individuals who have had bariatric surgery: 1. exhibit higher cognitive restraint and lower disinhibition and susceptibility to hunger, 2. eat more intuitively and, 3. have a less positive attitude towards weight gain.

## 2. Materials and Methods

### 2.1. Study Population

Data were collected in healthy pregnant volunteers from two different cohorts: CONFEC (CaractérisatiOn Nutritionnelle chez les Femmes Enceintes ayant subi une Chirurgie bariatrique) and ANGE (Apports Nutritionnels Durant la GrossessE). First, the CONFEC cohort [18] is composed of 40 pregnant individuals who underwent bariatric surgery and were recruited from May 2017 to August 2020 at the Centre Hospitalier Universitaire (CHU) de Québec-Université Laval. Briefly, the CONFEC study aimed to characterize dietary intakes at each trimester in association with dietary recommendations, GWG and fetal growth measurements. Individuals were included if they underwent bariatric surgery (any type) at the Institut Universitaire de Cardiologie et de Pneumologie de Québec (IUCPQ). They were excluded if they were younger than 18 years, had a twin or multiple pregnancy, a severe pre-existing medical condition (type 1 or type 2 diabetes, renal disease, inflammatory or autoimmune disorders) or were unable to consent. The final sample for this study totals 28 pregnant individuals (BPD-DS [n = 21] or SG [n = 7]). Second, the ANGE cohort (control group) is composed of 86 healthy pregnant individuals who were recruited from April 2016 to May 2017 at the CHU de Québec-Université Laval. The main objective of the ANGE study was to evaluate dietary intakes throughout pregnancy in association with dietary recommendations, GWG, glucose tolerance and metabolic profile. Detailed information on this study can be found elsewhere [11,19].

**Figure 1:**
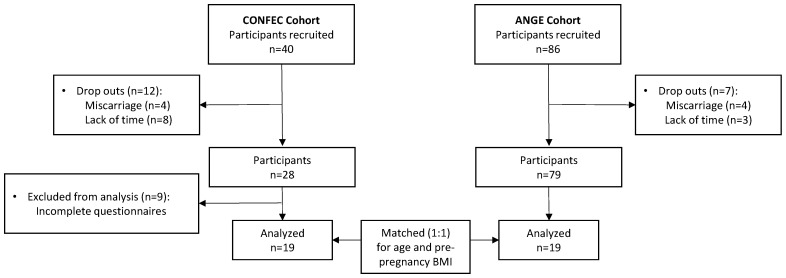
Flowchart of study population.

For the present study, we only included individuals from the CONFEC cohort who had successfully completed all the questionnaires outlined in the following sections (n = 19). Pregnant individuals from the CONFEC cohort were individually matched (1:1) for pre-pregnancy BMI and age, with the best match available from the ANGE cohort. After the matching process, the average differences observed between the pairs were 0.26 ± 1.18 kg/m^2^ for pre-pregnancy BMI and 0.38 ± 4.35 years for age (Figure 1). The final sample includes 19 participants in each group.

### 2.2. Ethics Statement

Both studies were conducted in line with the principles of the Declaration of Helsinki and were approved by the Ethics Committee of the Centre de recherche du *CHU de Québec-Université Laval* (CONFEC reference number: MP-20-2017-3217 and date of approval: 30 October 2016; ANGE reference number: 2016-2866 and date of approval: 7 March 2016). Informed consent was obtained from all individual participants included in the study.

### 2.3. Questionnaires

#### 2.3.1. Three-Factor Eating Questionnaire (TFEQ)

The TFEQ was completed by participants from both cohorts in the second trimester (CONFEC: 22.3 ± 1.2 weeks; ANGE: 22.3 ± 1.0 weeks). The TFEQ is a self-administered questionnaire containing 51 items, each of them allowing 0 or 1 point, and measuring three different eating behavior traits, known as factors: dietary restraint, disinhibition and susceptibility to hunger [20]. A higher TFEQ score reflects higher levels of dietary restraint, disinhibition, and susceptibility to hunger [20,21]. Total score and subscores are described below. The first factor, cognitive dietary restraint, can be described as a tendency to restrict food intake in order to control body weight [22]. Its score varies from 0 to 21 points. This factor includes two subscores: rigid restraint and flexible restraint. While rigid restraint is characterized by an all-or-nothing approach to eating, dieting, and weight [22], flexible restraint is characterized by a more gradual approach in which fattening foods are eaten in limited quantities without feelings of guilt [22]. The second factor, disinhibition, can be defined as overeating in response to different stimuli [23]. Its score varies from 0 to 16 points. This factor includes three subscores: habitual disinhibition (overeating in response to daily life circumstances [23]), emotional disinhibition (overeating in response to emotional states [23]) and situational disinhibition (overeating in response to specific environmental cues [23]). The third factor, susceptibility to hunger, can be defined as eating in response to perceived physiological symptoms [23]. Its score varies from 0 to 14 points. It includes 2 subscores: internal and external hunger which means that hunger can be regulated by internal or external cues [23].

#### 2.3.2. Intuitive Eating Scale 2 (IES-2)

The IES-2 was completed in the second trimester in both cohorts (CONFEC: 22.3 ± 1.2 weeks; ANGE: 22.3 ± 1.0 weeks). The IES-2 is a 23-item scale that measures a total intuitive eating score and 4 subscores: (1) unconditional permission to eat (6 items); (2) eating for physiological rather than emotional reasons (8 items); (3) reliance on internal hunger and satiety cues (6 items); (4) body–food choice congruence (3 items) [24,25]. Each item is rated on a five-point Likert scale ranging from “strongly disagree” (1 point) to “strongly agree” (5 points). Then, the mean scores for the total score and each of the 4 subscores are calculated. A higher score reflects a more intuitive way of eating [25]. This questionnaire was validated in French [25].

#### 2.3.3. Pregnancy Weight Gain Attitude Scale (PWGAS)

The French version of the PWGAS [26,27] was completed by participants during the third trimester (CONFEC: 32.2 ± 1.7 weeks; ANGE: 32.8 ± 0.6 weeks), since the French version was only validated in this specific trimester [11]. The original PWGAS is an 18-item questionnaire that assesses attitudes towards weight gain during pregnancy [27]. The French version of the PWGAS comprises 16 items (out of the 18 original items) [27]. It generates a total score and 5 subscores: (1) fear about weight gain (4 items); (2) absence of weight gain preoccupation (2 items); (3) positive attitudes about weight gain (4 items); (4) feeling overwhelmed by weight gain (3 items); (5) control over weight gain (3 items). This questionnaire also uses a five-point Likert scale, as described above. Mean scores for the total score and each of the 5 subscores were calculated. Higher scores reflect positive attitudes towards weight gain during pregnancy [27].

#### 2.3.4. Other Web-Based Questionnaires

Other web-based questionnaires were completed during the first trimester to collect information on medical history, previous pregnancies, tobacco use, education level and total household income.

### 2.4. Statistical Analyses

Matched pair analyses were used to compare the scores of all three questionnaires (i.e., TFEQ, IES-2 and PWGAS) between groups (matched for age and pre-pregnancy BMI). Continuous variables were compared using paired *t*-tests and are presented as means and standard deviations (SD). Differences between the paired measurements followed a normal distribution for all variables, with the exception of one, in which case a Wilcoxon signed-rank test was conducted. For categorical variables, Fisher’s exact tests were used to assess differences between the two groups. Post hoc analyses of the effect size were calculated for significant differences. *p* values < 0.05 were considered statistically significant. All statistical analyses were performed using JMP version 16 (SAS Institute Inc., Cary, NC, USA).

## 3. Results

### 3.1. Participant Characteristics

Table 1 presents the characteristics of participants. As expected, mean age and pre-pregnancy BMI were similar between individuals with or without a history of bariatric surgery. Additionally, both groups were similar for parity and total household income. However, participants with bariatric surgery had a significantly lower education level compared to participants without surgery (*p* = 0.004). Individuals with previous bariatric surgery had their surgery on average 4.8 ± 3.7 years before their current pregnancy.

### 3.2. Three-Factor Eating Questionnaire (TFEQ)

As shown in Table 2, no significant difference was found for each of the TFEQ scores in the second trimester. However, regarding subscores, pregnant individuals who had undergone surgery had a higher score for flexible restraint and a lower score for situational susceptibility to disinhibition compared to individuals without a history of bariatric surgery. No significant difference was observed for the other subscores measured by the TFEQ.

### 3.3. Intuitive Eating Scale (IES-2)

**Figure 2:**
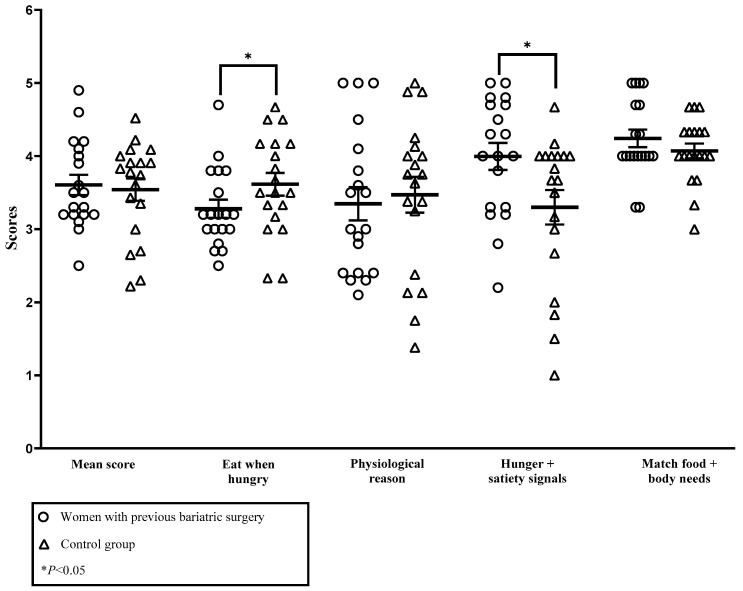
Comparison of the IES-2 (2nd trimester) mean score and subscores between pregnant individuals with or without an antecedent of bariatric surgery. Eat when hungry: unconditional permission to eat; physiological reason: eating for physiological rather than emotional reasons; Hunger + satiety signals: reliance on internal hunger and satiety cues; match food + body needs: body–food choice congruence. The effect size for unconditional permission to eat was 0.27 and 0.37 for reliance on internal hunger and satiety cues.

As shown in Figure 2, no significant difference was found for mean IES-2 score in the second trimester. However, pregnant individuals who had undergone surgery had a higher score for reliance on internal hunger and satiety cues subscore (3.99 ± 0.81 vs. 3.30 ± 1.03, *p* = 0.02) and a lower score for unconditional permission to eat subscore (3.28 ± 0.54 vs. 3.61 ± 0.68, *p* = 0.03). No significant difference was found for the other subscores of the IES-2.

### 3.4. Pregnancy Weight Gain Attitude Scale (PWGAS)

As shown in Table 3, no significant difference in attitudes towards weight gain was observed between groups in the third trimester.

## 4. Discussion

This study aimed to compare eating behaviors and attitudes towards food and weight gain between pregnant individuals with and without a history of bariatric surgery. Contrary to our hypotheses, no difference in global eating behaviors were found between study groups, as assessed using the total scores of the TFEQ, IES-2 and PWGAS questionnaires. Nevertheless, variations were identified in specific subscores of the utilized questionnaires. For the TFEQ, pregnant individuals with a history of bariatric surgery had a higher score for flexible restraint and a lower score for situational susceptibility to disinhibition compared to individuals with no history of bariatric surgery. Regarding the IES-2, pregnant individuals who have received surgery had a higher score for reliance on internal hunger and satiety cues and a lower one for unconditional permission to eat compared with those who had not received bariatric surgery. Very few, if any, comparable studies have been identified in the literature. As a result, comparisons with studies either post-bariatric surgery or during pregnancy will be made throughout the discussion.

Using data from the TFEQ, we did not observe any difference in total scores (cognitive restraint, disinhibition and susceptibility to hunger) between study groups. Since this is, to our knowledge, the first study to address this topic, some findings in related populations could provide some insight on our observations. First, in a non-pregnant population with a history of bariatric surgery, some studies have shown an increase in cognitive restraint and a decrease in hunger and disinhibition post bariatric surgery, as opposed to the present study [28,29,30,31,32]. Indeed, Konttinen et al. demonstrated that, compared with patients who have not received surgery, those who have received bariatric surgery (i.e., vertical banded gastroplasty and gastric bypass) experienced a significant decrease in susceptibility to hunger and disinhibition during the first year post surgery [33]. These changes tend to remain relatively stable in the following years. Regarding cognitive restriction, it rapidly increases during the first-year post-bariatric surgery but gradually decreases over time [33]. However, the changes in eating behaviors observed in these studies were seen in relatively short-term follow-ups (1–2 years) post-bariatric surgery [28,29,30,31,32,34] compared to our study where bariatric surgery was undertaken on average 5 years prior to the pregnancy. In brief, compared to literature on bariatric surgery non-pregnant patients, the absence of difference in TFEQ total scores between our two study groups could be partly explained by the time post-surgery.

Although we did not observe differences in the total scores of the eating behaviors studied, we found that pregnant individuals with a history of bariatric surgery had a higher score for flexible restraint and a lower score for situational susceptibility to disinhibition than those without bariatric surgery. On the one hand, this could partly be explained by the fact that, following bariatric surgery, satiety, food reward and preferences are modified [3]. Indeed, patients reduce their dietary intakes and modify their diet (i.e., avoid high-calorie foods and liquids, stop eating when feeling full, eat smaller amounts of food), but also adapt their eating behaviors to reduce postprandial discomfort in the short-term and to ensure no surgical complications in the long-term, especially when undergoing a surgery with a malabsorptive component [35]. It is also important to mention that, in the bariatric surgery population, suboptimal eating behaviors (e.g., restriction, binge eating) are common and may persist or even develop after surgery [36,37]. On the other hand, pregnant individuals with a history of bariatric surgery may have received more guidance, compared to pregnant individuals without surgery, because bariatric surgery pregnancies are considered high-risk in some settings. It has been demonstrated that personalized nutritional advice during post-bariatric surgery pregnancies improves the nutrient intake of mothers and can contribute to a higher birth weight for the baby [38]. Therefore, one could also think that an increased number of follow-ups could help identify eating behaviors that may be problematic in pregnancy (e.g., restriction due to fear of weight gain) and offer the necessary nutritional and psychological support. These factors could explain our results regarding the flexible restraint and situational disinhibition subscores. In summary of those findings, specific eating behaviors were different between groups, which could be explained by the physiological effects of surgery, the acquisition of new eating habits and the nutritional monitoring.

Our study highlighted that pregnant individuals with bariatric surgery have a higher score for reliance on internal hunger and satiety cues but a lower score for unconditional permission to eat than individuals without bariatric surgery. These results partially contradict our hypothesis that pregnant individuals with bariatric surgery are more intuitive when compared to pregnant individuals without bariatric surgery. First, to explain why pregnant individuals with bariatric surgery have higher score for reliance on internal hunger and satiety cues, we suggest that this may be related to the nutritional follow-up of these participants. Indeed, in order to minimize the risks of deficiencies and achieve adequate GWG, post bariatric surgery pregnant individuals are often guided and encouraged, throughout their pregnancy, to adopt a balanced diet specific to bariatric surgery and being attentive to their internal signals [15,39]. Second, changes in the gastrointestinal tract mean that certain foods are less tolerated or even discouraged to optimize intake and reduce the risk of inconvenience and/or discomfort [3]. This could thus explain why individuals who have undergone surgery do not have a high score for unconditional permission to eat. In brief, differences between pregnant individuals with and without bariatric surgery regarding intuitive eating were observed in this study, which could be explained by physiological changes and/or by the closer nutritional monitoring received throughout their pregnancy.

Furthermore, no difference in attitudes towards weight gain was observed between groups. This is in opposition to our hypothesis that pregnant individuals with bariatric surgery have a less positive attitude towards weight gain compared to pregnant non-bariatric surgery patients. This hypothesis stemmed from the rationale that since bariatric surgery is primarily intended for weight loss, any weight regain following the surgery may contribute to a more unfavorable outlook on weight gain within this group. A study by Meneguzzo et al. also suggests that a lack of improvement in body image in individuals following bariatric surgery may play a significant role in shaping their attitudes towards weight gain during pregnancy [40]. However, our results appear to demonstrate rather positive attitudes towards weight gain in late pregnancy in both groups (scores > 3 on average). Therefore, we can assume that pregnancy has a similar effect on attitudes towards weight gain in late pregnancy regardless of the history of bariatric surgery. In fact, in a pregnant population without a history of bariatric surgery, it has been shown that attitudes towards weight gain seem more positive in late pregnancy compared to the first trimester of pregnancy [41]. Furthermore, in a pregnant population with a history of bariatric surgery, literature shows significantly lower GWG compared to the population without a history of bariatric surgery, mainly in the third trimester [42], which may promote a less negative attitude towards weight gain in this population. In sum, the absence of differences between pregnant individuals with and without a history of bariatric surgery in attitudes towards weight gain could be explained by the fact that these elements were studied late during pregnancy and by the fact that GWG is typically minimal in post-bariatric surgery pregnancies.

To our knowledge, this is the first study to examine eating behaviors, intuitive eating components and attitudes towards weight gain and food in a population of pregnant individuals with bariatric surgery. Considering both total and subscores of various questionnaires allowed the identification of more specific differences between study groups that could not have been seen when only considering the total scores. Furthermore, the use of a matching method (1:1) for all analyses is a strength of this study, because age and BMI may influence the variables studied. However, we acknowledge that our matching method may have introduced a potential bias, as certain unmeasured characteristics might have influenced the selection of the “best match” for each participant. Furthermore, we could not match both cohorts for education level and household income as the ANGE cohort was too homogeneous (high socio-economic status), which represents a limitation of the current study. Indeed, education level and household income appear to have an impact on eating behaviors and attitudes towards weight gain [43,44]. As we found significant differences in education level between groups, we cannot exclude the possibility that education level may play a role in the differences we observed, suggesting a need for further analyses in a larger sample. Moreover, the CONFEC cohort includes two different types of surgery with an important variation of surgery-to-conception interval which may influence physiological changes and eating behaviors. The small sample size and its rather narrow representativeness may have reduced our statistical power and the generalizability of our data to other populations, as for example, the vast majority of the participants are Caucasian. Another limitation is that no data on whether or not the participants had seen a registered dietitian were collected. However, in the province of Québec, standard care typically involves scheduled consultations with a registered dietitian both prior to bariatric surgery and during pregnancy for individuals who have previously undergone such surgery. Further analyses are warranted in pregnant individuals who have a comparable socio-economic level, a similar type of surgery and a similar surgery-to-conception interval. Finally, our small sample size is explained by the many challenges encountered during the recruitment of this highly specific population, as well as losses due to follow-up or missing data. This project was entirely online, which may have diminished participant engagement. Other research efforts that include closer, possibly in-person, follow-up alongside their pregnancy monitoring could lead to a better understanding of the eating behaviors of this population.

## 5. Conclusions

Pregnant individuals with a history of bariatric surgery have some differences in eating behaviors and intuitive eating components when compared to pregnant individuals without a history of bariatric surgery. These differences must be confirmed among larger samples to better understand the implications of bariatric surgery on pregnancy and thus maximize the acquisition of healthy eating behaviors and attitudes towards weight gain in this population. In light of the present results, additional knowledge would enable healthcare professionals to better prevent and support these high-risk pregnancies by providing more personalized follow-up to help patients achieve optimal eating habits and attitudes towards weight gain. These findings underscore the need for confirmation through additional studies with larger sample sizes and more diverse populations to enhance the robustness and generalizability of the results.

## Figures and Tables

**Table 1:** Participants’ characteristics.

	Mean ± SD or N (%)		
Variables	Individuals with Previous Bariatric Surgery (n = 19)	Control Group (n = 19)	*p*-Value
Age (years)	32.7 ± 3.6	33.0 ± 3.6	0.71
Bariatric surgery			
BPD-DS	14 (74)		
LSG	5 (26)		
Surgery-to-conception interval (months)	57.5 ± 44.0		
≤18 months	4 (21)		
19–60 months	7 (38)		
>60 months	8 (43)		
Pre-pregnancy BMI (kg/m^2^)	31.2 ± 4.7	30.9 ± 5.1	0.35
Normal	2 (11)	1 (5)	
Overweight	5 (26)	8 (42)	
Obese	12 (63)	10 (53)	
Ethnicity (Caucasian)	14 (100) ^a^	18 (95)	
Nulliparity	5 (33) ^b^	9 (47)	0.50
Smokers	0 (0) ^c^	0 (0)	
Highest level of education			**0.0004**
Elementary	1 (7) ^b^	0 (0)	
High school	6 (40) ^b^	0 (0)	
College	3 (20) ^b^	2 (11)	
University	5 (33) ^b^	17 (89)	
Household income			0.93
CAD < 40,000	3 (21) ^a^	2 (11)	
CAD 40,000–59,999	2 (14) ^a^	3 (16)	
CAD 60,000–79,999	3 (21) ^a^	4 (21)	
CAD 80,000–99,999	2 (14) ^a^	5 (26)	
CAD > 100,000	4 (29) ^a^	5 (26)	

*p*-values refer to paired *t*-test or Fisher’s exact test; bold indicates statistically significant difference; BMI: body mass index; BPD-DS: biliopancreatic diversion with duodenal switch; SG: sleeve gastrectomy. ^a^ n = 14; ^b^ n = 15, ^c^ n = 13.

**Table 2:** Comparison of the Three Factor Eating Questionnaire’s (2nd trimester) total scores and subscores between pregnant individuals with or without an antecedent of bariatric surgery.

	Mean ± SD		
Variables (Variation in Scores)	Individuals with Previous Bariatric Surgery (n = 19)	Control Group (n = 19)	*p*-Value
Total restraint (0 to 21)	7.89 ± 3.26	7.11 ± 3.84	0.33
Rigid restraint (0 to 7)	2.00 ± 1.15	2.63 ± 1.77	0.17
Flexible restraint (0 to 7)	2.89 ± 1.15	1.95 ± 1.31	**0.04**
Total disinhibition (0 to 16)	5.53 ± 3.47	6.42 ± 2.99	0.36
Habitual disinhibition (0 to 5)	1.37 ± 1.46	1.00 ± 1.37	0.34
Emotional disinhibition (0 to 3)	1.42 ± 1.30	1.16 ± 1.17	0.50
Situational disinhibition (0 to 5)	1.11 ± 1.29	2.79 ± 1.44	**0.001**
Susceptibility to hunger (0 to 14)	4.74 ± 3.43	5.00 ± 2.92	0.82
Internal locus (0 to 6)	2.26 ± 1.85	2.00 ± 1.60	0.67
External locus (0 to 6)	1.47 ± 1.47	2.05 ± 1.72	0.27

*p*-values refer to paired *t*-test; bold indicates statistically significant difference. The effect size for flexible restraint was 0.38 and 0.61 for situational disinhibition.

**Table 3:** Comparison of the Pregnancy Weight Gain Attitude Scale questionnaire’s (3rd trimester) total score and subscores between pregnant individuals with or without an antecedent of bariatric surgery.

	Mean ± SD		
Variables	Individuals with Previous Bariatric Surgery (n = 19)	Control Group (n = 19)	*p*-Value
Total score	3.61 ± 0.50	3.79 ± 0.44	0.16
Fear about weight gain	3.57 ± 0.95	3.76 ± 0.71	0.45
Absence of weight gain preoccupation	3.68 ± 1.02	4.18 ± 0.93	0.10 ^a^
Positive attitudes about weight gain	3.25 ± 0.77	3.36 ± 0.75	0.69
Feeling overwhelmed by weight gain	4.42 ± 0.58	4.46 ± 0.61	0.84
Control over weight gain	3.28 ± 0.51	3.47 ± 0.88	0.33

*p*-values refer to paired *t*-test, except ^a^ refer to Wilcoxon signed rank.

## Data Availability

Data are contained within the article.
